# Comprehensive analysis of lncRNA-mediated ceRNA network in renal cell carcinoma based on GEO database

**DOI:** 10.1097/MD.0000000000039424

**Published:** 2024-08-30

**Authors:** Tianci Yang, Yixuan Li, Zhouhang Zheng, Pei Qu, Zhiang Shao, Jufang Wang, Nan Ding, Wei Wang

**Affiliations:** a The Second Clinical Medical School, Lanzhou University, Lanzhou, China; b Key Laboratory of Space Radiobiology of Gansu Province & Key Laboratory of Heavy Ion Radiation Biology and Medicine, Institute of Modern Physics, Chinese Academy of Sciences, Lanzhou, Gansu, China; c Department of Urology, Lanzhou University Second Hospital, Lanzhou, China.

**Keywords:** bioinformatics, competing endogenous RNAs network, long noncoding RNA, renal cell carcinoma, weighted gene co-expression network analysis

## Abstract

Renal cell carcinoma (RCC) ranks among the leading causes of cancer-related mortality. Despite extensive research, the precise etiology and progression of RCC remain incompletely elucidated. Long noncoding RNA (lncRNA) has been identified as competitive endogenous RNA (ceRNA) capable of binding to microRNA (miRNA) sites, thereby modulating the expression of messenger RNAs (mRNA) and target genes. This regulatory network is known to exert a pivotal influence on cancer initiation and progression. However, the specific role and functional significance of the lncRNA-miRNA-mRNA ceRNA network in RCC remain poorly understood. The RCC transcriptome data was obtained from the gene expression omnibus database. The identification of differentially expressed long noncoding RNAs (DElncRNAs), differentially expressed miRNAs, and differentially expressed mRNAs (DEmRNAs) between RCC and corresponding paracancer tissues was performed using the “Limma” package in R 4.3.1 software. We employed a weighted gene co-expression network analysis to identify the key DElncRNAs that are most relevant to RCC. Subsequently, we utilized the encyclopedia of RNA interactomes database to predict the interactions between these DElncRNAs and miRNAs, and the miRDB database to predict the interactions between miRNAs and mRNAs. Therefore, key DElncRNAs were obtained to verify the expression of their related genes in the The Cancer Genome Atlas database and to analyze the prognosis. The construction of RCC-specific lncRNA-miRNA-mRNA ceRNA network was carried out using Cytoscape 3.7.0. A total of 286 DElncRNAs, 56 differentially expressed miRNAs, and 2065 DEmRNAs were identified in RCC. Seven key DElncRNAs (GAS6 antisense RNA 1, myocardial infarction associated transcript, long intergenic nonprotein coding RNA 921, MMP25 antisense RNA 1, Chromosome 22 Open Reading Frame 34, MIR34A host gene, MIR4435-2 host gene) were identified using weighted gene co-expression network analysis and encyclopedia of RNA interactomes databases. Subsequently, a network diagram comprising 217 nodes and 463 edges was constructed based on these key DElncRNAs. The functional analysis of DEmRNAs in the ceRNA network was conducted using Kyoto Encyclopedia of Genes and Genomes and gene ontology. We constructed RCC-specific ceRNA networks and identified the crucial lncRNAs associated with RCC using bioinformatics analysis, which will help us further understand the pathogenesis of this disease.

## 1. Introduction

Renal cell carcinoma (RCC), also known as renal cancer, is a highly malignant tumor that occurs in the urinary system and is one of the most prevalent tumors, accounting for approximately 2% to 3% of all cases and comprising 80% to 90% of renal malignant tumors.^[1]^ The etiology of RCC remains incompletely understood, although smoking, obesity, hypertension, and chronic kidney disease have been identified as the most significant risk factors.^[2]^ Despite advancements in RCC treatment, its mortality rate remains elevated.^[3]^ Consequently, there is an urgent need to identify pivotal regulators which influence the malignancy of RCC.

Long noncoding RNA (lncRNA) refers to RNA molecules that exceed a length of 200 nucleotides and do not possess the ability to encode proteins.^[4]^ LncRNAs do not encode proteins, but can interact with proteins and nucleic acids that regulate gene expression in the nucleus and cytoplasm to establish stable and specific transcriptional and post transcriptional control.^[5]^ Recent investigations have highlighted the significant roles played by lncRNAs in various malignant tumors, encompassing diverse functions such as metabolic reprogramming, epithelial-interstitial transformation, and chromatin remodeling.^[6–8]^ The hypothesis that lncRNA functions as a competitive endogenous RNA (ceRNA) has garnered increased interest among various theories.^[9]^ ceRNA refers to a group of RNA molecules that can interact with microRNA (miRNA), thereby regulating the expression of other messenger RNAs (mRNAs) by competing for shared miRNA targets and forming gene expression networks.^[10,11]^ The ceRNA network has demonstrated significant involvement in the early diagnosis of hepatocellular carcinoma, tongue squamous cell carcinoma, glioblastoma, and various other malignancies.^[12–14]^ Recently, Wang Wei et al conducted experimental analysis and discovered that lncRNA UCA1 can act as a ceRNA to regulate the miR-182-5p/DLL4 axis, thereby promoting the malignant phenotype of RCC.^[15]^ Pritha Dasgupta also demonstrated that LncRNA CDKN2B-AS1/miR-141/cyclin D could regulate tumor progression and metastasis of renal cell carcinoma.^[16]^ However, previous studies have primarily focused on investigating the mechanism of individual lncRNA-miRNA-mRNA axes, and a RCC specific ceRNA network which uncover the key axes during RCC processes has yet to see.

**Figure 1.**
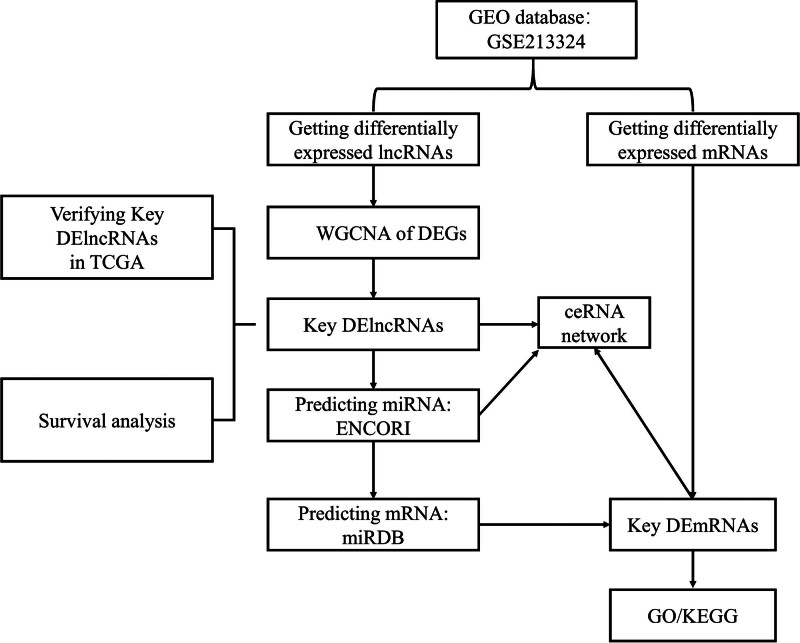
The workflow of ceRNA network study in RCC. ceRNA = competitive endogenous RNA.

This study aims to address this gap by employing the sangerbox tool and bioinformatics analysis to explore the functional mechanism of lncRNAs in RCC and construct RCC-specific ceRNA networks.^[17]^ First, the gene expression omnibus database (GEO) was used to obtain RCC related lncRNA, mRNA, and miRNA expression data. Then, we found differentially expressed genes (DEGs), differentially expressed miRNAs (DEmiRNAs) and differentially expressed long noncoding RNAs (DElncRNAs) using rstudio. Finally, the lncRNA-miRNA-mRNA ceRNA network was constructed using Cytoscape 3.7.0. The workflow diagram is shown in Figure 1.

## 2. Materials and methods

### 2.1. Data collection and processing

RNAseq data, lncRNA sequence data and corresponding RCC clinical data were downloaded from the GEO database GSE213324 (https://www.ncbi.nlm.nih.gov/geo/query/acc.cgi?acc=GSE213324). A total of 63 renal cancer tissue samples and 60 normal renal cancer tissue samples were collected. The original expression matrix was normalized and the DEGs were identified by limma R package. Limma, which stands for linear models for microarray data, is a statistical method that utilizes generalized linear models for identifying DEGs.^[18]^ In this study, we employed the R software package limma (version 3.40.6) to conduct differential analysis in order to identify genes that exhibit differential expression between various comparison groups and control groups. To begin, we applied a log2 transformation to the expression spectrum dataset and subsequently performed multiple linear regression using the lmFit function. We then utilized the eBays function to compute moderated t-statistics, moderated F-statistics, and log-odds of differential expression through empirical Bayes moderation of the standard errors towards a common value. Ultimately, we obtained the significance of each gene.

### 2.2. WGCNA of DEGs

In order to enhance the understanding of the involvement of DElncRNAs in RCC, we constructed the weighted gene co-expression network analysis (WGCNA) network.^[19]^ Initially, we calculated the median absolute deviation for each gene based on the profile of DEGs. Subsequently, we excluded the top 0% of genes with the lowest median absolute deviation and employed the goodSamplesGenes function from the R software package WGCNA to eliminate aberrant genes and samples. Furthermore, we utilized WGCNA to establish a scale-free co-expression network. Initially, the Pearson correlation matrix and average linking method were employed to assess the relationship between all pairs of genes. Subsequently, a weighted adjacency matrix was constructed using a power function, denoted as A_mn=|C_mn|^β^, where C_mn represents the Pearson correlation between Gene_m and Gene_n, and A_mn represents the adjacency between Gene_m and Gene_n. The parameter β served as a soft-thresholding factor, enabling the amplification of robust correlations among genes while penalizing weaker correlations. After selecting a power of 22, the adjacency was converted into a topological overlap matrix (TOM) to assess the network connectivity of a gene, defined as the sum of its adjacency with all other genes for network gene ratio. The corresponding dissimilarity (1-TOM) was then computed. To categorize genes with similar expression profiles into gene modules, average linkage hierarchical clustering was performed using the TOM-based dissimilarity measure, with a minimum size (gene group) of 30 for the genes dendrogram. The sensitivity was set to 3. In order to conduct a more comprehensive analysis of the module, we performed dissimilarity calculations on the eigen Genes of the module, determined a cut line for the module dendrogram, and merged certain modules. Furthermore, we integrated modules with a distance below 0.25, resulting in the identification of 11 co-expression modules. It is worth noting that gray module is considered to be a gene set that cannot be assigned to any module.

### 2.3. Obtain key DElncRNAs

We conducted a correlation analysis between modules and clinical features, aiming to identify modules that are most relevant to tumors. To achieve this, we calculated the correlation between gene expression and modules to obtain gene significance (GS) values. Additionally, we calculated the correlation between Module feature vectors and gene expression to obtain module membership (MM) values. By applying the cutoff criteria (|MM| > 0.8 and |GS| > 0.1), we successfully identified 1017 genes with high connectivity in clinically significant modules. Furthermore, we screened out all differentially expressed lncrnas in the corresponding module, which were deemed as key DElncRNAs.

### 2.4. Expression of key DElncRNAs in different pathological types of kidney cancer

The standardized pan-cancer dataset from the UCSC database (https://xenabrowser.net/), specifically the The Cancer Genome Atlas (TCGA) Pan-Cancer (PANCAN, N = 10535, G = 60499), was obtained. From this dataset, the expression data of the hub gene in various tissue types of renal cancer were extracted. Subsequently, the sample sources were screened to include Solid Tissue Normal, Primary Blood Derived Cancer-Peripheral Blood, and Primary Tumor samples. To normalize the expression values, a logarithmic transformation of log2(x + 0.001) was applied. The expression data of kidney chromophobe (KICH), pan-kidney cohort (KICH + KIRC + KIRP) (KIPAN), kidney renal clear cell carcinoma (KIRC), and kidney renal papillary cell carcinoma (KIRP) were subjected to screening. The disparity in expression levels between normal and tumor samples for each type of tumor was determined using R software (version 3.6.4), and the statistical significance of the differences was assessed through an unpaired Student *t* Test.

### 2.5. Prognostic analysis of key DElncRNAs in RCC

The optimum cutoff value was determined through the utilization of the R package maxstat (version: 0.7-25), which employs maximally selected rank statistics with various *P*-value approximations. To establish the optimal truncation value for DElncRNAs, the minimum sample number was set to exceed 25% while the maximum sample number was limited to <75%. Subsequently, patients were stratified into high and low groups based on the obtained optimal cutoff value. The survfit function from the survival package in R software was employed to examine the prognostic disparities between the 2 groups. The logrank test was utilized to assess the significance of prognostic differences among samples from distinct groups. The outcomes were visualized as a Kaplan–Meier curve.

### 2.6. Analysis of miRNAs-lncRNAs and miRNAs-mRNAs interaction network

The encyclopedia of RNA interactomes (ENCORI) database was utilized to forecast the miRNAs that interact with key DElncRNAs. Subsequently, 2 or more miRNAs that interacted with DElncRNAs were identified as the key miRNAs. The miRDB database was employed to predict the mRNAs that regulate these key miRNAs, with a target score threshold set at >90. The intersection of these predicted mRNAs with the differentially expressed mRNAs (DEmRNAs) in GSE213324 was considered as the core mRNAs. Lastly, adhering to the ceRNA hypothesis, a ceRNA network was constructed and the outcomes were visually represented using Cytoscape (version 3.7.0).

### 2.7. Enrichment analysis

For the purpose of conducting mRNA enrichment analysis, we utilized the Kyoto Encyclopedia of Genes and Genomes (KEGG) rest API (https://www.kegg.jp/kegg/rest/keggapi.html) and the Gene GO (gene ontology) annotation from the R package. In order to establish the background set, the genes were mapped accordingly. Subsequently, the enrichment analysis was performed using the R software package clusterProfiler (version 3.14.3) to obtain the enrichment results of the gene set. The minimum gene set size was defined as 5, while the maximum gene set size was set at 5000. Statistical significance was determined by a *P* value of <.05 and a FDR (false discovery rate) of <0.1.

## 3. Results

### 3.1. Identification of DEGs in renal cell carcinoma

**Figure 2.**
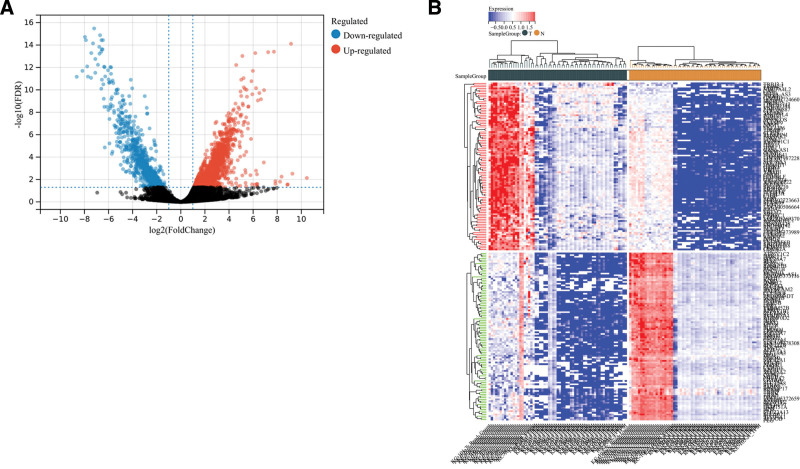
Identification of differentially expressed lncRNAs in RCC and normal tissues. (A) The volcano plot showed that a total of 2682 upregulated lncRNAs and 938 downregulated lncRNAs were screened out; (B) heat map of whole gene differential expression. lncRNAs = long noncoding RNA, RCC = renal cell carcinoma.

We conducted an analysis of differential genes in a cohort of 63 RCC samples and 60 normal renal tissue samples. Using a significance threshold of FDR < 0.05 and |log fold change| > 2, we identified a total of 3620 RCC-specific differential genes, comprising 286 DElncRNAs, 56 DEmiRNAs, and 2065 DEmRNAs. These differential genes were visualized in a volcano map (Fig. 2A) and a heat map (Fig. 2B). In the volcano map, up-regulated gene markers are represented by a light red color, while down-regulated genes are labeled in blue. The criteria for differential gene selection were |fold change| > 2 and FDR < 0.05.

### 3.2. Construction of gene co-expression module

**Figure 3.**
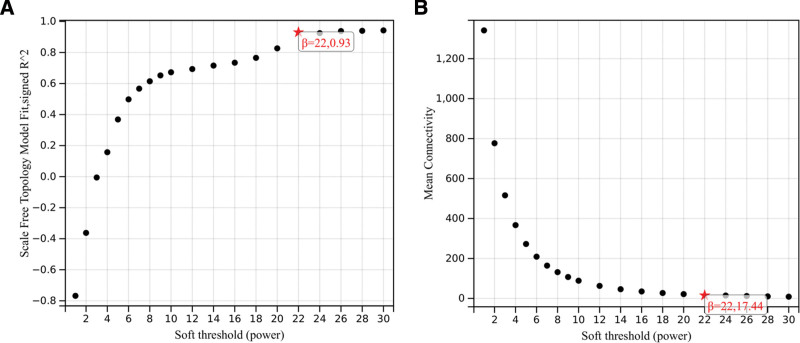
Determination of soft-threshold power in the WGCNA. (A) Analysis of the scale-free index for various soft-threshold powers (β). (B) Analysis of the mean connectivity for various soft-threshold powers. WGCNA = weighted gene co-expression network analysis.

To establish the WGCNA network, the initial step involves determining the soft threshold power β and enhancing the coexpression similarity for adjacency calculation. This process is executed through the utilization of the pickSoftThreshold function within the WGCNA framework, which facilitates the examination of network topology. Subsequently, a soft threshold power β of 22 is selected for further analysis, as it attains a scale independence of 0.9 (Fig. 3A) and exhibits a relatively high mean connectivity (Fig. 3B).

**Figure 4.**
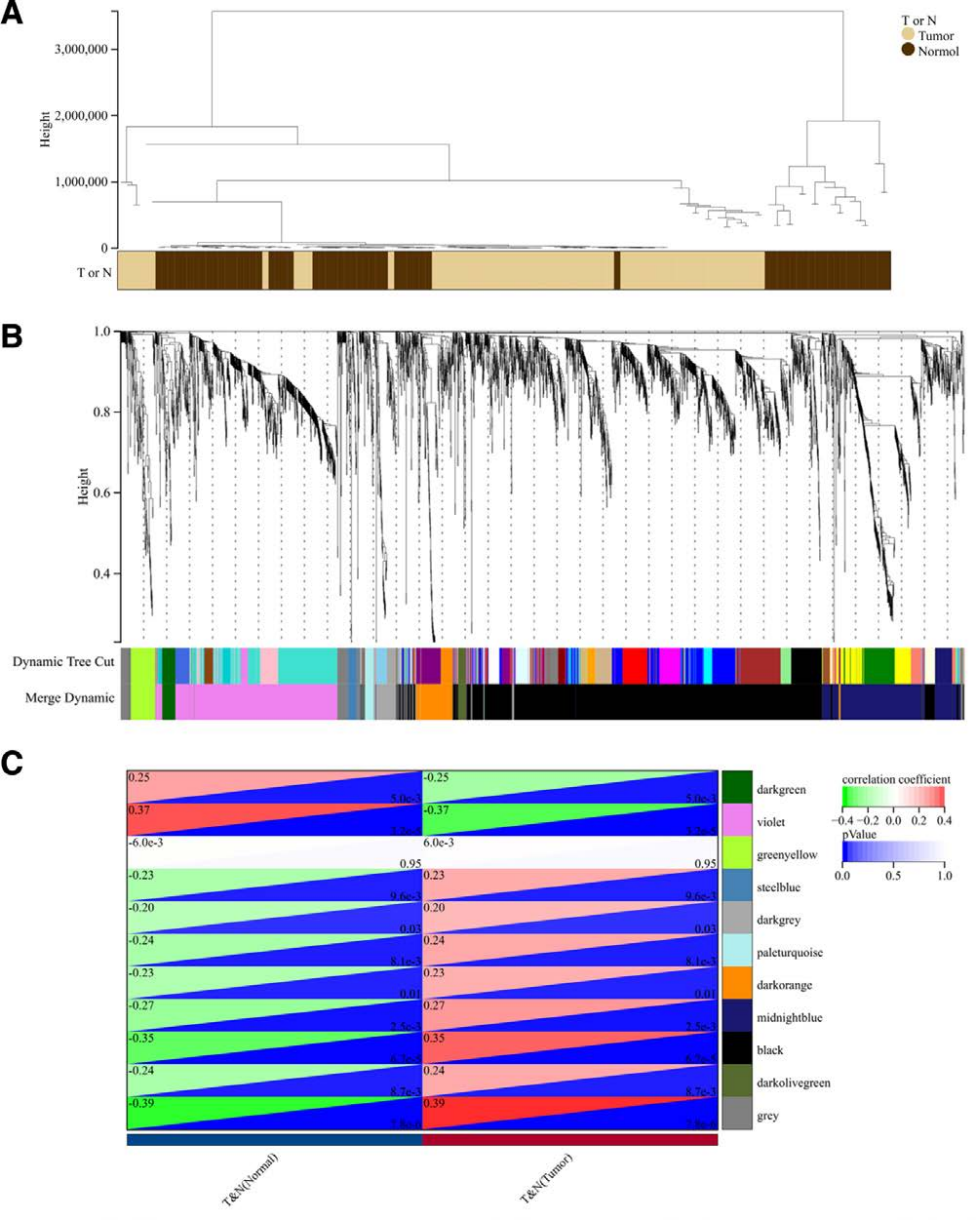
WGCNA key steps. (A) Clustering dendrogram of 123 samples. (B) Dendrogram of all differentially expressed genes clustered based on the measurement of dissimilarity (1-TOM). The color band shows the results obtained from the automatic single-block analysis. (C) Heatmap of the correlation between the module eigengenes and clinical traits of RCC. We selected the black-Tumor block for subsequent analysis. TOM = topological overlap matrix, WGCNA = weighted gene co-expression network analysis.

The construction of gene networks and identification of modules are accomplished using the one-step network building capability provided by the WGCNA R package. In the process of cluster splitting, the soft threshold power was established at 22, the minimum module size was defined as 30, and a high sensitivity of 3 was employed. Subsequently, a total of 11 gene co-expression modules were successfully constructed (Figure 4A and B). These modules were then examined for correlations with clinical features, with a focus on identifying significant associations. The findings revealed that the black modules exhibited the strongest association with renal cell carcinoma (Fig. 4C).

### 3.3. Acquisition of key DElncRNAs

Based on the criteria of GS >0.1 and MM >0.8, a total of 1017 genes within the black module were identified as key genes, which contains 63 DElncRNAs. Among these, 7 DElncRNAs (GAS6 antisense RNA 1 [GAS6-AS1], myocardial infarction associated transcript [MIAT], long intergenic nonprotein coding RNA 921 [LINC00921], MMP25 antisense RNA 1 [MMP25-AS1], Chromosome 22 Open Reading Frame 34 [C22orf34], MIR34A host gene [MIR34AHG], MIR4435-2 host gene [MIR4435-2HG]) were retrieved from the ENCORI database and recognized as the final hub genes. Notably, these DElncARNAs exhibited significant upregulation in the GSE213324 dataset.

### 3.4. Verify the expression of key DElncRNAs in different pathological types of kidney cancer

**Figure 5.**
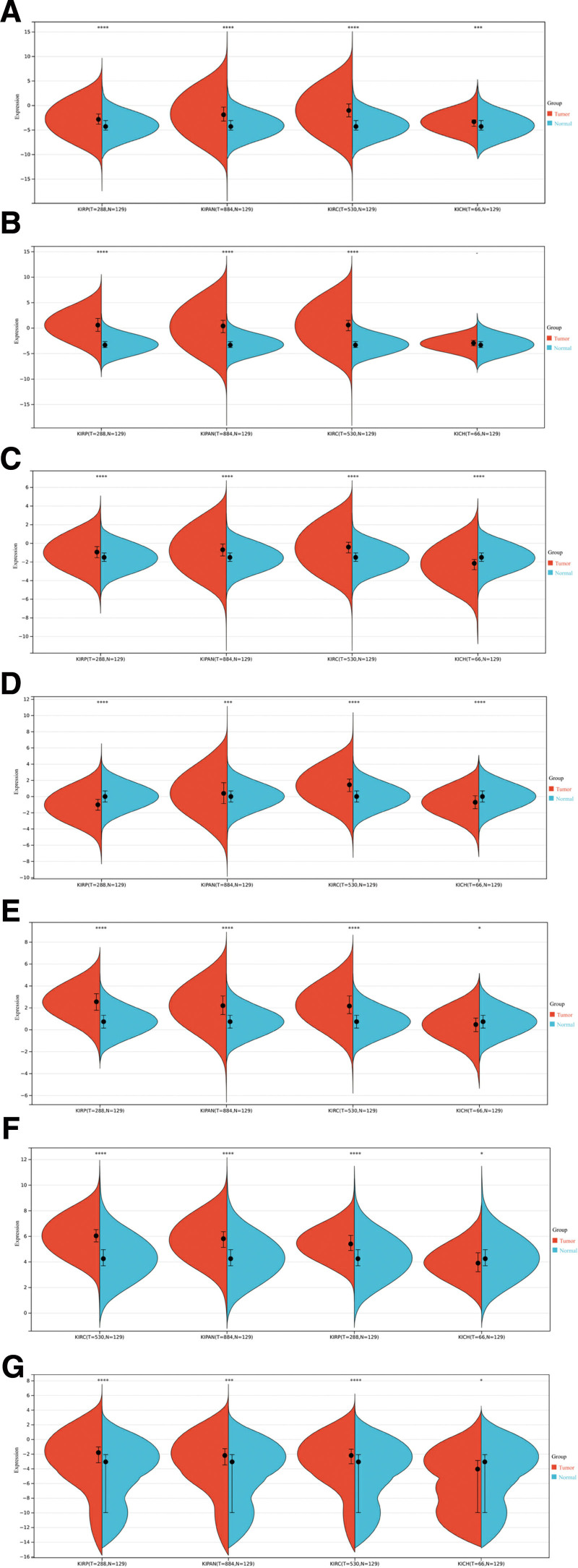
Expression of key DElncRNAs in different pathological types of renal carcinoma in TCGA database. (A) MIAT, (B) GAS6-AS1, (C) LINC00921, (D) C22orf34, (E) MMP25-AS1, (F) MIR4435-2HG, (G) MIR34AHG. DElncRNAs = differentially expressed long noncoding RNAs, RCC = renal cell carcinoma.

The TCGA database analysis revealed the differential expression of 7 DElncRNAs namely GAS6-AS1, MIAT, LINC00921, MMP25-AS1, C22orf34, MIR34AHG, and MIR4435-2HG in 4 distinct pathological subtypes of renal cancer (Fig. 5). Our findings indicate a significant up-regulation of these 7 DElncRNAs in KIPAN, KIRC, and KIRP subtypes. In the KICH subtype, MIAT and GAS6-AS1 were found to be up-regulated, although the difference was not statistically significant. Conversely, the remaining 5 DElncRNAs exhibited a significant down-regulation in the KICH subtype. The aforementioned findings indicate that the expression patterns of the 7 DElncRNAs identified from the GSE213324 dataset in the GEO database were largely in agreement with those observed in the TCGA database for RCC. With the exception of KICH, a distinct pathological subtype of renal cancer, the identified key DElncRNAs exhibited elevated expression levels in various other types of renal cancer.

### 3.5. Prognostic analysis of key DElncRNAs in RCC

**Figure 6.**
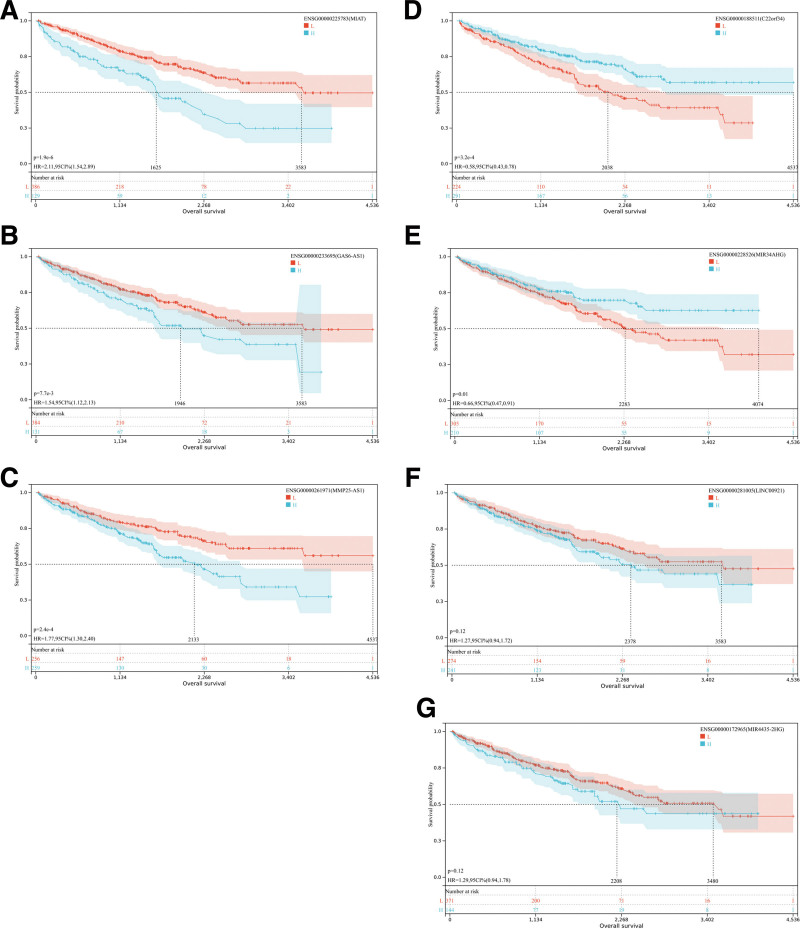
Kaplan–Meier curve analysis of key DElncRNAs for the overall survival in RCC patients. (A) MIAT, (B) GAS6-AS1, (C) MMP25-AS1, (D) C22orf34, (E) MIR34AHG, (F) LINC00921, (G) MIR4435-2HG. DElncRNAs = differentially expressed long noncoding RNAs.

RCC patients were stratified into high and low groups based on the optimal cutoff value, and the prognostic difference between these groups was assessed using the logrank test. The survival analysis results indicate that GAS6-AS1, MIAT, and MMP25-AS1 exhibit significant disparities in the overall survival of RCC patients. Specifically, the group with high expression of GAS6-AS1, MIAT, and MMP25-AS1 demonstrated a lower overall survival rate compared to the group with low and high expression of these genes. There existed notable disparities in the overall survival of RCC patients between C22orf34 and MIR34AHG. The cohort with elevated expression levels of both C22orf34 and MIR34AHG exhibited a superior overall survival rate compared to the cohort with diminished expression levels of C22orf34 and MIR34AHG. The presence of LINC00921 and MIR4435-2HG did not yield any substantial impact on the overall survival of RCC patients. All of the above results are shown in Figure 6.

### 3.6. Construction and analysis of lncRNA-miRNA-mRNA ceRNA network

**Figure 7.**
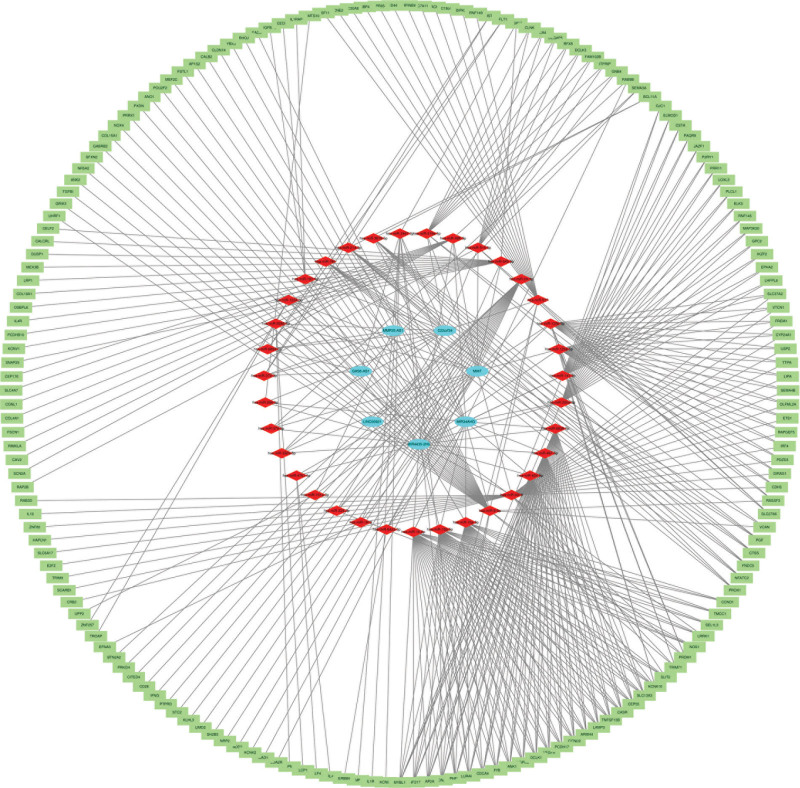
lncRNA-miRNA-mRNA ceRNA network in RCC. Oval, diamond and rectangle represent DElncRNAs, DEmiRNAs, and DEmRNAs, respectively. ceRNA = competitive endogenous RNA. DElncRNA = differentially expressed lncRNA, DEmiRNA = differentially expressed miRNA, DEmRNA = differentially expressed mRNAs.

The ENCORI database was utilized to forecast the miRNAs that interacted with key DElncRNAs. From this analysis, a total of 36 miRNAs were identified to interact with 2 or more key DElncRNAs, as presented in Table S1, Supplemental Digital Content, http://links.lww.com/MD/N483. The miRDB database was employed to predict the mRNAs that regulate these miRNAs, with a target score threshold set at >90. The resulting intersection was obtained by comparing with DEmRNAs in GSE213324, and the findings are displayed in Table S2, Supplemental Digital Content, http://links.lww.com/MD/N483. Ultimately, a network diagram comprising 217 nodes and 463 edges was constructed, visualizing the interactions among lncRNAs, miRNAs, and mRNAs using Cytoscape, as depicted in Figure 7.

### 3.7. Functional enrichment analysis of DEmRNAs

**Figure 8.**
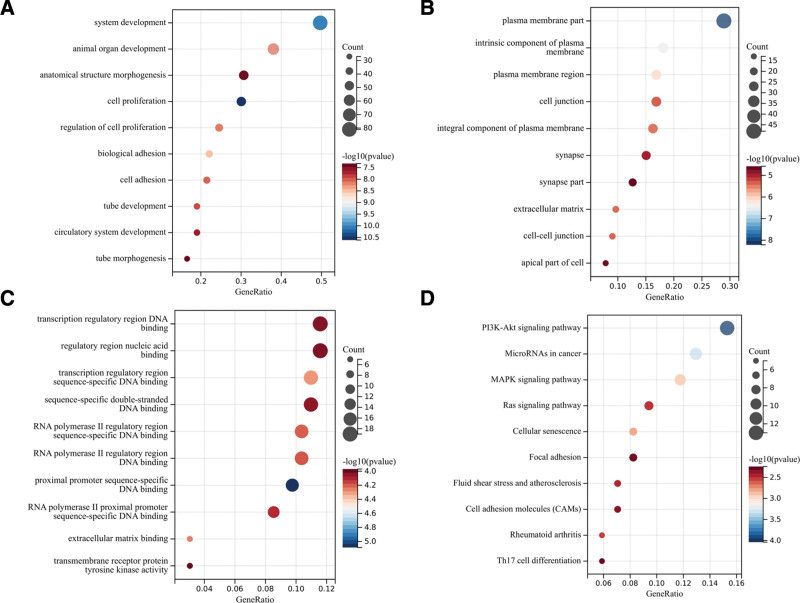
GO and KEGG enrichment analysis of DEmRNAs in the ceRNA network. (A) Bubble plot of BP. (B) Bubble plot of CC. (C) Bubble plot of MF. (D) Bubble plot of KEGG. BP = biological processes; CC = cell component; ceRNA = competitive endogenous RNA; GO = gene ontology; KEGG = Kyoto Encyclopedia of Genes and Genomes; MF = molecular function.

The findings from the GO analysis demonstrated that the DEmRNAs were significantly enriched in various biological processes, including cell proliferation, system development, biological adhesion, regulation of cell proliferation, animal organ development, cell adhesion, tube development, circulatory system development, anatomical structure morphogenesis, and tube morphogenesis (Fig. 8A).The changes of cell composition were mainly concentrated in plasma membrane part, intrinsic component of plasma membrane, plasma membrane region, integral component of plasma membrane, extracellular matrix, cell–cell junction, cell junction, synapse, apical part of cell, synapse part (Fig. 8B).The changes of molecular function are mainly concentrated in proximal promoter sequence-specific, DNA binding, transcription regulatory region sequence-specific DNA binding, extracellular matrix binding, RNA polymerase II regulatory region sequence-specific DNA binding, RNA polymerase II regulatory region DNA binding, RNA polymerase II proximal promoter sequence-specific DNA binding, sequence-specific double-stranded DNA binding, transcription regulatory region DNA binding, regulatory region nucleic acid binding, transmembrane receptor protein tyrosine kinase activity (Fig. 8C). KEGG pathway analysis showed that DEmRNAs were mainly enriched in PI3K-Akt signaling pathway, microRNAs in cancer, MAPK signaling pathway, cellular senescence, rheumatoid arthritis, Ras signaling pathway, fluid shear stress and atherosclerosis, cell adhesion molecules, focal adhesion, Th17 cell differentiation, proteoglycans in cancer, Rap1 signaling pathway, p53 signaling pathway (Fig. 8D).

## 4. Discussion

RCC ranks among the leading causes of cancer-related mortality, yet its pathogenesis remains incompletely elucidated.^[20]^ RCC is fundamentally a metabolic disorder distinguished by the reprogramming of energy metabolism.^[21–24]^ In particular the metabolic flux through glycolysis is partitioned,^[25–27]^ and mitochondrial bioenergetics and OxPhox are impaired, as well as lipid metabolism.^[25,28–31]^ In this context, lncRNA and miRNA have been demonstrated to serve as pivotal regulatory factors in cell metabolism, modulating various biological attributes of renal cancer stem cells.^[32]^ In addition, renal cell carcinoma is one of the most immune-infiltrated tumors.^[33–35]^ Emerging evidence suggests that the activation of specific metabolic pathway have a role in regulating angiogenesis and inflammatory signatures.^[36,37]^ Features of the tumor microenvironment heavily affect disease biology and may affect responses to systemic therapy.^[38–40]^ lncRNAs can modulate immune cell infiltration and regulate immunoflogosisThe ceRNA hypothesis introduces a novel regulatory mechanism facilitated by lncRNAs, wherein they function as endogenous miRNA decoys.^[41]^ In this study, a total of 63 kidney cancer samples and 60 normal kidney cancer tissue samples were collected from the GEO database GSE213324 dataset. Through analysis, we identified a total of 3620 RCC-specific differential genes, which included 286 DElncRNAs, 56 DEmiRNAs, and 2065 DEmRNAs. The WGCNA method was employed to screen out 63 DElncRNAs that exhibited a close association with RCC. Furthermore, the ENCORI database was utilized to identify 7 key DElncRNAs, namely GAS6-AS1, MIAT, LINC00921, MMP25-AS1, C22orf34, MIR34AHG, and MIR4435-2HG. The miRNAs interacting with these DElncRNAs were also predicted. The interaction between the miRNAs and DElncRNAs was predicted, along with the prediction of mRNAs that regulate these miRNAs using the miRDB database and their intersection with the DEmRNAs in GSE213324. The ceRNA network was visualized using Cytoscape. Furthermore, we conducted verification in the TCGA database, which revealed that key DElncRNAs were highly expressed in the major pathological subtypes of 3 types of renal carcinoma (KIPAN, KIRC, KIRP). Notably, GAS6-AS1, MIAT, MMP25-AS1, C22orf34, and MIR34AHG exhibited significant differences in the overall survival of RCC patients. The group characterized by high expression of GAS6-AS1, MIAT, and MMP25-AS1 exhibited a significantly lower overall survival rate compared to the group with low expression of these genes.

LncRNA GAS6-AS1 has been highly expressed in gastric cancer, hepatocellular carcinoma, breast cancer and acute myeloid leukemia. However, the precise role of GAS6-AS1 in tumorigenesis remains incompletely elucidated.^[42]^ Qing Chen et al demonstrated that GAS6-AS1 regulates Tripartite Motif Containing 14 through sponge miR-370-3p/miR-1296-5p to participate in accelerating the growth and metastasis of colorectal cancer.^[43]^ The aforementioned pathway bears resemblance to the GAS6-AS1/miR-370-3p/fms related receptor tyrosine kinase 1 axis within our anticipated ceRNA network specific to RCC. Fms related receptor tyrosine kinase 1, akin to Tripartite Motif Containing 14, assumes the role of a regulator in the NF-κB signaling pathway,^[44,45]^ and the aberrant functioning of NF-κB activity is implicated in the pathogenesis of inflammation-related ailments and malignancies.^[46]^

LncRNA MIAT was originally discovered in a genome-wide association study of Japanese patients with myocardial infarction.^[47]^ MIAT has been found to act as a sponge for miR-150-5p, thereby contributing to the development of thyroid, cervical, ovarian, and diabetes-induced microvascular dysfunction.^[48–51]^ Furthermore, it has been reported that the MIAT-miR-324-3p-LASP1 pathway plays a significant role in promoting the proliferation and invasion of thyroid cancer, with its activation being closely associated with the inactivation of PI3K/AKT signal transduction.^[52]^

There is limited literature available on LncRNA MMP25-AS1. However, it is noteworthy that Peng Tan et al conducted a bioinformatics analysis and identified the MMP25-AS1/hsa-miR-10a-5p/Serpin Family E Member 1 axis as a novel prognostic biomarker associated with immune cell infiltration in KIRC.^[53]^ This pathway has the potential to modulate the expression of chemokines (CCL4, CCL5, CXCL13, and X-C Motif Chemokine Ligand 2), thereby influencing the tumor immune microenvironment and the development of KIRC. These findings have significant implications for enhancing our understanding of the role of MMP25-AS1 in RCC.

The present study examined the functional role of the aforementioned 3 lncRNAs as ceRNAs in various types of cancers, demonstrating a significant concordance with the findings from KEGG and GO databases. Gene enrichment analyses revealed the crucial involvement of pathways such as PI3K-Akt signaling, microRNAs in cancer, cell proliferation, and regulation of cell proliferation in tumor progression.^[54–57]^

This study has certain limitations, primarily stemming from the exclusive reliance on data obtained from public databases. Moreover, the dataset we screened did not distinguish TNM stage of RCC patients. Consequently, the findings derived from bioinformatics analysis merely offer insights into the pathogenesis of RCC. To ascertain the molecular mechanism of ceRNA in RCC progression, it is imperative to conduct additional cell and animal experiments in future research endeavors.

## 5. Conclusion

We employed bioinformatics analysis to construct ceRNA networks specific to RCC and identified key long noncoding RNAs (lncRNAs) associated with RCC. Our study presents a novel approach for the identification of potential lncRNA biomarkers. Furthermore, the elucidation of the ceRNA network in RCC enhances our comprehension of the underlying mechanisms contributing to the development and progression of this disease.

## Acknowledgments

We are grateful for the multiple databases that provide very useful data.

## Author contributions

**Formal analysis:** Pei Qu, Zhiang Shao.

**Funding acquisition:** Wei Wang.

**Methodology:** Yixuan Li, Zhouhang Zheng.

**Supervision:** Jufang Wang, Nan Ding, Wei Wang.

**Writing – original draft:** Tianci Yang.

**Writing – review & editing:** Nan Ding.

## Supplementary Material


