# Supplementary material for: Comprehensive analysis of lncRNA-mediated ceRNA network in renal cell carcinoma based on GEO database
Source: Medicine (Baltimore). 2024 Aug 30;103(35):e39424. doi: 10.1097/MD.0000000000039424 (PMC11365686; doi:10.1097/MD.0000000000039424)
Supplement: Supplementary file 1 [file medi-103-e39424-s001.docx]

Supplementary Table 1: Predicting miRNAs interacting with key DElncRNAs using ENCORI database

| Target | Type | miRNA | Type |
| --- | --- | --- | --- |
| GAS6-AS1 | lncRNA | hsa-miR-370-3p | miRNA |
| GAS6-AS1 | lncRNA | hsa-miR-483-3p | miRNA |
| GAS6-AS1 | lncRNA | hsa-miR-2467-3p | miRNA |
| GAS6-AS1 | lncRNA | hsa-miR-6893-3p | miRNA |
| MIAT | lncRNA | hsa-miR-642a-5p | miRNA |
| MIAT | lncRNA | hsa-miR-4761-5p | miRNA |
| MIAT | lncRNA | hsa-miR-5586-5p | miRNA |
| MIAT | lncRNA | hsa-miR-24-3p | miRNA |
| MIAT | lncRNA | hsa-miR-1913 | miRNA |
| MIAT | lncRNA | hsa-miR-324-3p | miRNA |
| MIAT | lncRNA | hsa-miR-1224-5p | miRNA |
| MIAT | lncRNA | hsa-miR-151a-3p | miRNA |
| MIAT | lncRNA | hsa-miR-125b-5p | miRNA |
| MIAT | lncRNA | hsa-miR-125a-5p | miRNA |
| MIAT | lncRNA | hsa-miR-4319 | miRNA |
| MIAT | lncRNA | hsa-miR-515-5p | miRNA |
| MIAT | lncRNA | hsa-miR-519e-5p | miRNA |
| MIAT | lncRNA | hsa-miR-488-3p | miRNA |
| MIAT | lncRNA | hsa-miR-545-3p | miRNA |
| MIAT | lncRNA | hsa-miR-3529-5p | miRNA |
| MIAT | lncRNA | hsa-miR-379-5p | miRNA |
| LINC00921 | lncRNA | hsa-miR-9-5p | miRNA |
| LINC00921 | lncRNA | hsa-miR-214-3p | miRNA |
| MMP25-AS1 | lncRNA | hsa-miR-761 | miRNA |
| MMP25-AS1 | lncRNA | hsa-miR-3619-5p | miRNA |
| MMP25-AS1 | lncRNA | hsa-miR-197-3p | miRNA |
| MMP25-AS1 | lncRNA | hsa-miR-296-5p | miRNA |
| C22orf34 | lncRNA | hsa-miR-577 | miRNA |
| C22orf34 | lncRNA | hsa-miR-141-3p | miRNA |
| C22orf34 | lncRNA | hsa-miR-200a-3p | miRNA |
| C22orf34 | lncRNA | hsa-miR-15a-5p | miRNA |
| C22orf34 | lncRNA | hsa-miR-16-5p | miRNA |
| C22orf34 | lncRNA | hsa-miR-15b-5p | miRNA |
| C22orf34 | lncRNA | hsa-miR-424-5p | miRNA |
| C22orf34 | lncRNA | hsa-miR-6838-5p | miRNA |
| C22orf34 | lncRNA | hsa-miR-497-5p | miRNA |
| C22orf34 | lncRNA | hsa-miR-24-3p | miRNA |
| C22orf34 | lncRNA | hsa-miR-151a-3p | miRNA |
| C22orf34 | lncRNA | hsa-miR-6893-3p | miRNA |
| C22orf34 | lncRNA | hsa-miR-370-3p | miRNA |
| C22orf34 | lncRNA | hsa-miR-483-3p | miRNA |
| C22orf34 | lncRNA | hsa-miR-2467-3p | miRNA |
| C22orf34 | lncRNA | hsa-miR-324-3p | miRNA |
| MIR34AHG | lncRNA | hsa-miR-1913 | miRNA |
| MIR34AHG | lncRNA | hsa-miR-519e-5p | miRNA |
| MIR34AHG | lncRNA | hsa-miR-515-5p | miRNA |
| MIR34AHG | lncRNA | hsa-miR-370-3p | miRNA |
| MIR34AHG | lncRNA | hsa-miR-6893-3p | miRNA |
| MIR34AHG | lncRNA | hsa-miR-5586-5p | miRNA |
| MIR34AHG | lncRNA | hsa-miR-379-5p | miRNA |
| MIR34AHG | lncRNA | hsa-miR-3529-5p | miRNA |
| MIR34AHG | lncRNA | hsa-miR-642a-5p | miRNA |
| MIR34AHG | lncRNA | hsa-miR-2467-3p | miRNA |
| MIR34AHG | lncRNA | hsa-miR-3619-5p | miRNA |
| MIR34AHG | lncRNA | hsa-miR-214-3p | miRNA |
| MIR34AHG | lncRNA | hsa-miR-761 | miRNA |
| MIR34AHG | lncRNA | hsa-miR-488-3p | miRNA |
| MIR34AHG | lncRNA | hsa-miR-1224-5p | miRNA |
| MIR34AHG | lncRNA | hsa-miR-9-5p | miRNA |
| MIR34AHG | lncRNA | hsa-miR-296-5p | miRNA |
| MIR4435-2HG | lncRNA | hsa-miR-9-5p | miRNA |
| MIR4435-2HG | lncRNA | hsa-miR-296-5p | miRNA |
| MIR4435-2HG | lncRNA | hsa-miR-4761-5p | miRNA |
| MIR4435-2HG | lncRNA | hsa-miR-1224-5p | miRNA |
| MIR4435-2HG | lncRNA | hsa-miR-497-5p | miRNA |
| MIR4435-2HG | lncRNA | hsa-miR-16-5p | miRNA |
| MIR4435-2HG | lncRNA | hsa-miR-424-5p | miRNA |
| MIR4435-2HG | lncRNA | hsa-miR-6838-5p | miRNA |
| MIR4435-2HG | lncRNA | hsa-miR-15a-5p | miRNA |
| MIR4435-2HG | lncRNA | hsa-miR-15b-5p | miRNA |
| MIR4435-2HG | lncRNA | hsa-miR-545-3p | miRNA |
| MIR4435-2HG | lncRNA | hsa-miR-577 | miRNA |
| MIR4435-2HG | lncRNA | hsa-miR-197-3p | miRNA |
| MIR4435-2HG | lncRNA | hsa-miR-4319 | miRNA |
| MIR4435-2HG | lncRNA | hsa-miR-125b-5p | miRNA |
| MIR4435-2HG | lncRNA | hsa-miR-125a-5p | miRNA |
| MIR4435-2HG | lncRNA | hsa-miR-488-3p | miRNA |
| MIR4435-2HG | lncRNA | hsa-miR-379-5p | miRNA |
| MIR4435-2HG | lncRNA | hsa-miR-3529-5p | miRNA |
| MIR4435-2HG | lncRNA | hsa-miR-141-3p | miRNA |
| MIR4435-2HG | lncRNA | hsa-miR-200a-3p | miRNA |

Supplementary Table 2: Predicting the mRNAs which regulate these miRNAs using the miRDB database

| miRNA | Type | Target | Type |
| --- | --- | --- | --- |
| hsa-miR-370-3p | miRNA | CHST11 | mRNA |
| hsa-miR-370-3p | miRNA | FLT1 | mRNA |
| hsa-miR-2467-3p | miRNA | KCNE4 | mRNA |
| hsa-miR-2467-3p | miRNA | IL1RN | mRNA |
| hsa-miR-2467-3p | miRNA | MMP16 | mRNA |
| hsa-miR-2467-3p | miRNA | USP37 | mRNA |
| hsa-miR-2467-3p | miRNA | LURAP1L | mRNA |
| hsa-miR-2467-3p | miRNA | PHF19 | mRNA |
| hsa-miR-6893-3p | miRNA | CHST11 | mRNA |
| hsa-miR-6893-3p | miRNA | FLT1 | mRNA |
| hsa-miR-642a-5p | miRNA | ERBB4 | mRNA |
| hsa-miR-4761-5p | miRNA | ARL4C | mRNA |
| hsa-miR-4761-5p | miRNA | KLF4 | mRNA |
| hsa-miR-5586-5p | miRNA | LCP1 | mRNA |
| hsa-miR-5586-5p | miRNA | LRRK1 | mRNA |
| hsa-miR-5586-5p | miRNA | PTPN12 | mRNA |
| hsa-miR-24-3p | miRNA | EDA2R | mRNA |
| hsa-miR-24-3p | miRNA | GAD1 | mRNA |
| hsa-miR-24-3p | miRNA | KCNK2 | mRNA |
| hsa-miR-24-3p | miRNA | ADD2 | mRNA |
| hsa-miR-24-3p | miRNA | NRP2 | mRNA |
| hsa-miR-24-3p | miRNA | SH2B3 | mRNA |
| hsa-miR-24-3p | miRNA | LIMD2 | mRNA |
| hsa-miR-24-3p | miRNA | KLHL3 | mRNA |
| hsa-miR-24-3p | miRNA | STC2 | mRNA |
| hsa-miR-24-3p | miRNA | PTPRD | mRNA |
| hsa-miR-24-3p | miRNA | IFNG | mRNA |
| hsa-miR-24-3p | miRNA | CD28 | mRNA |
| hsa-miR-24-3p | miRNA | CITED4 | mRNA |
| hsa-miR-24-3p | miRNA | PRKCH | mRNA |
| hsa-miR-24-3p | miRNA | BTN2A2 | mRNA |
| hsa-miR-1913 | miRNA | EFNA3 | mRNA |
| hsa-miR-324-3p | miRNA | NOS1 | mRNA |
| hsa-miR-1224-5p | miRNA | TROAP | mRNA |
| hsa-miR-1224-5p | miRNA | ZNF257 | mRNA |
| hsa-miR-151a-3p | miRNA | UPP2 | mRNA |
| hsa-miR-125b-5p | miRNA | SLC37A2 | mRNA |
| hsa-miR-125b-5p | miRNA | VTCN1 | mRNA |
| hsa-miR-125b-5p | miRNA | FREM1 | mRNA |
| hsa-miR-125b-5p | miRNA | KCNK10 | mRNA |
| hsa-miR-125b-5p | miRNA | PRDM1 | mRNA |
| hsa-miR-125b-5p | miRNA | CYP24A1 | mRNA |
| hsa-miR-125b-5p | miRNA | USP2 | mRNA |
| hsa-miR-125b-5p | miRNA | TTPA | mRNA |
| hsa-miR-125b-5p | miRNA | LIPA | mRNA |
| hsa-miR-125b-5p | miRNA | TRIM71 | mRNA |
| hsa-miR-125b-5p | miRNA | SEMA4B | mRNA |
| hsa-miR-125b-5p | miRNA | OLFML2A | mRNA |
| hsa-miR-125b-5p | miRNA | ETS1 | mRNA |
| hsa-miR-125b-5p | miRNA | RAPGEF5 | mRNA |
| hsa-miR-125b-5p | miRNA | IRF4 | mRNA |
| hsa-miR-125b-5p | miRNA | PDZD3 | mRNA |
| hsa-miR-125b-5p | miRNA | DIRAS1 | mRNA |
| hsa-miR-125b-5p | miRNA | CDH5 | mRNA |
| hsa-miR-125a-5p | miRNA | SLC37A2 | mRNA |
| hsa-miR-125a-5p | miRNA | VTCN1 | mRNA |
| hsa-miR-125a-5p | miRNA | FREM1 | mRNA |
| hsa-miR-125a-5p | miRNA | KCNK10 | mRNA |
| hsa-miR-125a-5p | miRNA | PRDM1 | mRNA |
| hsa-miR-125a-5p | miRNA | CYP24A1 | mRNA |
| hsa-miR-125a-5p | miRNA | USP2 | mRNA |
| hsa-miR-125a-5p | miRNA | TTPA | mRNA |
| hsa-miR-125a-5p | miRNA | LIPA | mRNA |
| hsa-miR-125a-5p | miRNA | TRIM71 | mRNA |
| hsa-miR-125a-5p | miRNA | SEMA4B | mRNA |
| hsa-miR-125a-5p | miRNA | OLFML2A | mRNA |
| hsa-miR-125a-5p | miRNA | ETS1 | mRNA |
| hsa-miR-125a-5p | miRNA | RAPGEF5 | mRNA |
| hsa-miR-125a-5p | miRNA | IRF4 | mRNA |
| hsa-miR-125a-5p | miRNA | PDZD3 | mRNA |
| hsa-miR-125a-5p | miRNA | DIRAS1 | mRNA |
| hsa-miR-125a-5p | miRNA | CDH5 | mRNA |
| hsa-miR-4319 | miRNA | SLC37A2 | mRNA |
| hsa-miR-4319 | miRNA | CRB2 | mRNA |
| hsa-miR-4319 | miRNA | VTCN1 | mRNA |
| hsa-miR-4319 | miRNA | SCARB1 | mRNA |
| hsa-miR-4319 | miRNA | FREM1 | mRNA |
| hsa-miR-4319 | miRNA | KCNK10 | mRNA |
| hsa-miR-4319 | miRNA | PRDM1 | mRNA |
| hsa-miR-4319 | miRNA | TRIM9 | mRNA |
| hsa-miR-4319 | miRNA | E2F2 | mRNA |
| hsa-miR-4319 | miRNA | CYP24A1 | mRNA |
| hsa-miR-4319 | miRNA | USP2 | mRNA |
| hsa-miR-4319 | miRNA | TTPA | mRNA |
| hsa-miR-4319 | miRNA | SLC6A17 | mRNA |
| hsa-miR-4319 | miRNA | LIPA | mRNA |
| hsa-miR-4319 | miRNA | TRIM71 | mRNA |
| hsa-miR-4319 | miRNA | HAPLN1 | mRNA |
| hsa-miR-4319 | miRNA | ZNF80 | mRNA |
| hsa-miR-4319 | miRNA | SEMA4B | mRNA |
| hsa-miR-4319 | miRNA | OLFML2A | mRNA |
| hsa-miR-4319 | miRNA | ETS1 | mRNA |
| hsa-miR-4319 | miRNA | RAPGEF5 | mRNA |
| hsa-miR-4319 | miRNA | IL16 | mRNA |
| hsa-miR-4319 | miRNA | IRF4 | mRNA |
| hsa-miR-4319 | miRNA | PDZD3 | mRNA |
| hsa-miR-4319 | miRNA | DIRAS1 | mRNA |
| hsa-miR-4319 | miRNA | RAB3D | mRNA |
| hsa-miR-4319 | miRNA | CDH5 | mRNA |
| hsa-miR-515-5p | miRNA | CLNK | mRNA |
| hsa-miR-515-5p | miRNA | CA4 | mRNA |
| hsa-miR-515-5p | miRNA | DLGAP5 | mRNA |
| hsa-miR-515-5p | miRNA | RFX8 | mRNA |
| hsa-miR-515-5p | miRNA | RASSF3 | mRNA |
| hsa-miR-515-5p | miRNA | DCLK3 | mRNA |
| hsa-miR-515-5p | miRNA | RAP2B | mRNA |
| hsa-miR-515-5p | miRNA | FAM102B | mRNA |
| hsa-miR-515-5p | miRNA | ITPRIP | mRNA |
| hsa-miR-515-5p | miRNA | GNB4 | mRNA |
| hsa-miR-515-5p | miRNA | SLC27A6 | mRNA |
| hsa-miR-519e-5p | miRNA | CLNK | mRNA |
| hsa-miR-519e-5p | miRNA | CA4 | mRNA |
| hsa-miR-519e-5p | miRNA | DLGAP5 | mRNA |
| hsa-miR-519e-5p | miRNA | RFX8 | mRNA |
| hsa-miR-519e-5p | miRNA | RASSF3 | mRNA |
| hsa-miR-519e-5p | miRNA | DCLK3 | mRNA |
| hsa-miR-519e-5p | miRNA | FAM102B | mRNA |
| hsa-miR-519e-5p | miRNA | ITPRIP | mRNA |
| hsa-miR-519e-5p | miRNA | GNB4 | mRNA |
| hsa-miR-519e-5p | miRNA | SLC27A6 | mRNA |
| hsa-miR-488-3p | miRNA | SCN2A | mRNA |
| hsa-miR-488-3p | miRNA | CAV2 | mRNA |
| hsa-miR-488-3p | miRNA | RIMKLA | mRNA |
| hsa-miR-488-3p | miRNA | FSCN1 | mRNA |
| hsa-miR-488-3p | miRNA | COL4A1 | mRNA |
| hsa-miR-488-3p | miRNA | CGNL1 | mRNA |
| hsa-miR-488-3p | miRNA | RAB9B | mRNA |
| hsa-miR-488-3p | miRNA | SLC4A7 | mRNA |
| hsa-miR-488-3p | miRNA | CEP170 | mRNA |
| hsa-miR-545-3p | miRNA | SNAP25 | mRNA |
| hsa-miR-545-3p | miRNA | KCNV1 | mRNA |
| hsa-miR-545-3p | miRNA | PCDHB10 | mRNA |
| hsa-miR-545-3p | miRNA | IL4R | mRNA |
| hsa-miR-545-3p | miRNA | OSBPL6 | mRNA |
| hsa-miR-545-3p | miRNA | COL19A1 | mRNA |
| hsa-miR-545-3p | miRNA | LRP1 | mRNA |
| hsa-miR-545-3p | miRNA | MEX3B | mRNA |
| hsa-miR-545-3p | miRNA | DUSP1 | mRNA |
| hsa-miR-545-3p | miRNA | CALCRL | mRNA |
| hsa-miR-545-3p | miRNA | CELF2 | mRNA |
| hsa-miR-545-3p | miRNA | USP37 | mRNA |
| hsa-miR-3529-5p | miRNA | SEMA3A | mRNA |
| hsa-miR-379-5p | miRNA | SEMA3A | mRNA |
| hsa-miR-9-5p | miRNA | UHRF1 | mRNA |
| hsa-miR-9-5p | miRNA | GRIK3 | mRNA |
| hsa-miR-9-5p | miRNA | TGFBI | mRNA |
| hsa-miR-9-5p | miRNA | ANK2 | mRNA |
| hsa-miR-9-5p | miRNA | PRDM1 | mRNA |
| hsa-miR-9-5p | miRNA | NR5A2 | mRNA |
| hsa-miR-9-5p | miRNA | VCAN | mRNA |
| hsa-miR-9-5p | miRNA | SFXN2 | mRNA |
| hsa-miR-9-5p | miRNA | KCNJ2 | mRNA |
| hsa-miR-9-5p | miRNA | GABRB2 | mRNA |
| hsa-miR-9-5p | miRNA | COL15A1 | mRNA |
| hsa-miR-9-5p | miRNA | NOX4 | mRNA |
| hsa-miR-9-5p | miRNA | PRRX1 | mRNA |
| hsa-miR-9-5p | miRNA | PXDN | mRNA |
| hsa-miR-9-5p | miRNA | ANO1 | mRNA |
| hsa-miR-9-5p | miRNA | POU2F2 | mRNA |
| hsa-miR-9-5p | miRNA | RASSF3 | mRNA |
| hsa-miR-9-5p | miRNA | MEF2C | mRNA |
| hsa-miR-9-5p | miRNA | TRIM71 | mRNA |
| hsa-miR-9-5p | miRNA | FSTL1 | mRNA |
| hsa-miR-9-5p | miRNA | AP1S2 | mRNA |
| hsa-miR-9-5p | miRNA | CALB2 | mRNA |
| hsa-miR-9-5p | miRNA | CLDN14 | mRNA |
| hsa-miR-9-5p | miRNA | YBX3 | mRNA |
| hsa-miR-9-5p | miRNA | RHOJ | mRNA |
| hsa-miR-9-5p | miRNA | SACS | mRNA |
| hsa-miR-214-3p | miRNA | PGF | mRNA |
| hsa-miR-214-3p | miRNA | CTSS | mRNA |
| hsa-miR-214-3p | miRNA | FNDC5 | mRNA |
| hsa-miR-214-3p | miRNA | BCL11A | mRNA |
| hsa-miR-214-3p | miRNA | NFATC2 | mRNA |
| hsa-miR-214-3p | miRNA | PROX1 | mRNA |
| hsa-miR-761 | miRNA | PGF | mRNA |
| hsa-miR-761 | miRNA | CTSS | mRNA |
| hsa-miR-761 | miRNA | FNDC5 | mRNA |
| hsa-miR-761 | miRNA | NFATC2 | mRNA |
| hsa-miR-761 | miRNA | PROX1 | mRNA |
| hsa-miR-3619-5p | miRNA | PGF | mRNA |
| hsa-miR-3619-5p | miRNA | CTSS | mRNA |
| hsa-miR-3619-5p | miRNA | FNDC5 | mRNA |
| hsa-miR-3619-5p | miRNA | BCL11A | mRNA |
| hsa-miR-3619-5p | miRNA | NFATC2 | mRNA |
| hsa-miR-3619-5p | miRNA | PROX1 | mRNA |
| hsa-miR-197-3p | miRNA | IGFBP3 | mRNA |
| hsa-miR-197-3p | miRNA | CECR2 | mRNA |
| hsa-miR-197-3p | miRNA | IL1RAP | mRNA |
| hsa-miR-296-5p | miRNA | ADAMTS10 | mRNA |
| hsa-miR-577 | miRNA | IGSF11 | mRNA |
| hsa-miR-577 | miRNA | CCNE2 | mRNA |
| hsa-miR-577 | miRNA | TFAP2A | mRNA |
| hsa-miR-577 | miRNA | SLC30A8 | mRNA |
| hsa-miR-577 | miRNA | RBP4 | mRNA |
| hsa-miR-577 | miRNA | GPR65 | mRNA |
| hsa-miR-577 | miRNA | CD44 | mRNA |
| hsa-miR-577 | miRNA | SERPINB9 | mRNA |
| hsa-miR-577 | miRNA | SLC7A11 | mRNA |
| hsa-miR-577 | miRNA | DACH1 | mRNA |
| hsa-miR-577 | miRNA | C18orf54 | mRNA |
| hsa-miR-577 | miRNA | DIPK2A | mRNA |
| hsa-miR-577 | miRNA | SLC27A6 | mRNA |
| hsa-miR-577 | miRNA | RNF149 | mRNA |
| hsa-miR-577 | miRNA | RNF217 | mRNA |
| hsa-miR-141-3p | miRNA | VCAN | mRNA |
| hsa-miR-141-3p | miRNA | MYBL1 | mRNA |
| hsa-miR-141-3p | miRNA | GJC1 | mRNA |
| hsa-miR-141-3p | miRNA | ELMOD1 | mRNA |
| hsa-miR-141-3p | miRNA | CSTA | mRNA |
| hsa-miR-141-3p | miRNA | PAQR9 | mRNA |
| hsa-miR-141-3p | miRNA | JAZF1 | mRNA |
| hsa-miR-141-3p | miRNA | P2RY1 | mRNA |
| hsa-miR-141-3p | miRNA | PRR11 | mRNA |
| hsa-miR-141-3p | miRNA | LOXL3 | mRNA |
| hsa-miR-141-3p | miRNA | PLCL1 | mRNA |
| hsa-miR-141-3p | miRNA | ELK3 | mRNA |
| hsa-miR-141-3p | miRNA | RNF145 | mRNA |
| hsa-miR-141-3p | miRNA | MAP3K20 | mRNA |
| hsa-miR-141-3p | miRNA | GPC2 | mRNA |
| hsa-miR-141-3p | miRNA | IKZF2 | mRNA |
| hsa-miR-141-3p | miRNA | EPHA2 | mRNA |
| hsa-miR-141-3p | miRNA | LHFPL6 | mRNA |
| hsa-miR-200a-3p | miRNA | VCAN | mRNA |
| hsa-miR-200a-3p | miRNA | MYBL1 | mRNA |
| hsa-miR-200a-3p | miRNA | GJC1 | mRNA |
| hsa-miR-200a-3p | miRNA | ELMOD1 | mRNA |
| hsa-miR-200a-3p | miRNA | CSTA | mRNA |
| hsa-miR-200a-3p | miRNA | PAQR9 | mRNA |
| hsa-miR-200a-3p | miRNA | JAZF1 | mRNA |
| hsa-miR-200a-3p | miRNA | P2RY1 | mRNA |
| hsa-miR-200a-3p | miRNA | PRR11 | mRNA |
| hsa-miR-200a-3p | miRNA | LOXL3 | mRNA |
| hsa-miR-200a-3p | miRNA | PLCL1 | mRNA |
| hsa-miR-200a-3p | miRNA | ELK3 | mRNA |
| hsa-miR-200a-3p | miRNA | RNF145 | mRNA |
| hsa-miR-200a-3p | miRNA | MAP3K20 | mRNA |
| hsa-miR-200a-3p | miRNA | GPC2 | mRNA |
| hsa-miR-200a-3p | miRNA | IKZF2 | mRNA |
| hsa-miR-200a-3p | miRNA | EPHA2 | mRNA |
| hsa-miR-200a-3p | miRNA | LHFPL6 | mRNA |
| hsa-miR-15a-5p | miRNA | SLC13A3 | mRNA |
| hsa-miR-15a-5p | miRNA | CEP55 | mRNA |
| hsa-miR-15a-5p | miRNA | TFAP2A | mRNA |
| hsa-miR-15a-5p | miRNA | CASR | mRNA |
| hsa-miR-15a-5p | miRNA | NOS1 | mRNA |
| hsa-miR-15a-5p | miRNA | KCNK10 | mRNA |
| hsa-miR-15a-5p | miRNA | TNFSF13B | mRNA |
| hsa-miR-15a-5p | miRNA | MYBL1 | mRNA |
| hsa-miR-15a-5p | miRNA | KCNJ2 | mRNA |
| hsa-miR-15a-5p | miRNA | LAMP3 | mRNA |
| hsa-miR-15a-5p | miRNA | ARMH4 | mRNA |
| hsa-miR-15a-5p | miRNA | CCND2 | mRNA |
| hsa-miR-15a-5p | miRNA | PCDH17 | mRNA |
| hsa-miR-15a-5p | miRNA | LRRK1 | mRNA |
| hsa-miR-15a-5p | miRNA | VEGFA | mRNA |
| hsa-miR-15a-5p | miRNA | SLIT2 | mRNA |
| hsa-miR-15a-5p | miRNA | DCLK1 | mRNA |
| hsa-miR-15a-5p | miRNA | CCND1 | mRNA |
| hsa-miR-15a-5p | miRNA | HSPG2 | mRNA |
| hsa-miR-15a-5p | miRNA | TRANK1 | mRNA |
| hsa-miR-15a-5p | miRNA | TMCC1 | mRNA |
| hsa-miR-15a-5p | miRNA | SEL1L3 | mRNA |
| hsa-miR-15a-5p | miRNA | LURAP1L | mRNA |
| hsa-miR-15a-5p | miRNA | MYB | mRNA |
| hsa-miR-15a-5p | miRNA | RNF217 | mRNA |
| hsa-miR-15a-5p | miRNA | PHF19 | mRNA |
| hsa-miR-15a-5p | miRNA | CDCA4 | mRNA |
| hsa-miR-16-5p | miRNA | SLC13A3 | mRNA |
| hsa-miR-16-5p | miRNA | CEP55 | mRNA |
| hsa-miR-16-5p | miRNA | TFAP2A | mRNA |
| hsa-miR-16-5p | miRNA | CASR | mRNA |
| hsa-miR-16-5p | miRNA | NOS1 | mRNA |
| hsa-miR-16-5p | miRNA | KCNK10 | mRNA |
| hsa-miR-16-5p | miRNA | TNFSF13B | mRNA |
| hsa-miR-16-5p | miRNA | MYBL1 | mRNA |
| hsa-miR-16-5p | miRNA | KCNJ2 | mRNA |
| hsa-miR-16-5p | miRNA | LAMP3 | mRNA |
| hsa-miR-16-5p | miRNA | ARMH4 | mRNA |
| hsa-miR-16-5p | miRNA | CCND2 | mRNA |
| hsa-miR-16-5p | miRNA | PCDH17 | mRNA |
| hsa-miR-16-5p | miRNA | LRRK1 | mRNA |
| hsa-miR-16-5p | miRNA | VEGFA | mRNA |
| hsa-miR-16-5p | miRNA | SLIT2 | mRNA |
| hsa-miR-16-5p | miRNA | DCLK1 | mRNA |
| hsa-miR-16-5p | miRNA | CCND1 | mRNA |
| hsa-miR-16-5p | miRNA | RAB9B | mRNA |
| hsa-miR-16-5p | miRNA | HSPG2 | mRNA |
| hsa-miR-16-5p | miRNA | TRANK1 | mRNA |
| hsa-miR-16-5p | miRNA | TMCC1 | mRNA |
| hsa-miR-16-5p | miRNA | SEL1L3 | mRNA |
| hsa-miR-16-5p | miRNA | LURAP1L | mRNA |
| hsa-miR-16-5p | miRNA | MYB | mRNA |
| hsa-miR-16-5p | miRNA | RNF217 | mRNA |
| hsa-miR-16-5p | miRNA | PHF19 | mRNA |
| hsa-miR-16-5p | miRNA | CDCA4 | mRNA |
| hsa-miR-15b-5p | miRNA | SLC13A3 | mRNA |
| hsa-miR-15b-5p | miRNA | CEP55 | mRNA |
| hsa-miR-15b-5p | miRNA | TFAP2A | mRNA |
| hsa-miR-15b-5p | miRNA | CASR | mRNA |
| hsa-miR-15b-5p | miRNA | NOS1 | mRNA |
| hsa-miR-15b-5p | miRNA | KCNK10 | mRNA |
| hsa-miR-15b-5p | miRNA | TNFSF13B | mRNA |
| hsa-miR-15b-5p | miRNA | MYBL1 | mRNA |
| hsa-miR-15b-5p | miRNA | KCNJ2 | mRNA |
| hsa-miR-15b-5p | miRNA | LAMP3 | mRNA |
| hsa-miR-15b-5p | miRNA | ARMH4 | mRNA |
| hsa-miR-15b-5p | miRNA | CCND2 | mRNA |
| hsa-miR-15b-5p | miRNA | PCDH17 | mRNA |
| hsa-miR-15b-5p | miRNA | LRRK1 | mRNA |
| hsa-miR-15b-5p | miRNA | VEGFA | mRNA |
| hsa-miR-15b-5p | miRNA | SLIT2 | mRNA |
| hsa-miR-15b-5p | miRNA | DCLK1 | mRNA |
| hsa-miR-15b-5p | miRNA | CCND1 | mRNA |
| hsa-miR-15b-5p | miRNA | HSPG2 | mRNA |
| hsa-miR-15b-5p | miRNA | TRANK1 | mRNA |
| hsa-miR-15b-5p | miRNA | TMCC1 | mRNA |
| hsa-miR-15b-5p | miRNA | SEL1L3 | mRNA |
| hsa-miR-15b-5p | miRNA | LURAP1L | mRNA |
| hsa-miR-15b-5p | miRNA | MYB | mRNA |
| hsa-miR-15b-5p | miRNA | RNF217 | mRNA |
| hsa-miR-15b-5p | miRNA | PHF19 | mRNA |
| hsa-miR-15b-5p | miRNA | CDCA4 | mRNA |
| hsa-miR-424-5p | miRNA | SLC13A3 | mRNA |
| hsa-miR-424-5p | miRNA | CEP55 | mRNA |
| hsa-miR-424-5p | miRNA | TFAP2A | mRNA |
| hsa-miR-424-5p | miRNA | CASR | mRNA |
| hsa-miR-424-5p | miRNA | TNFSF13B | mRNA |
| hsa-miR-424-5p | miRNA | MYBL1 | mRNA |
| hsa-miR-424-5p | miRNA | KCNJ2 | mRNA |
| hsa-miR-424-5p | miRNA | LAMP3 | mRNA |
| hsa-miR-424-5p | miRNA | ARMH4 | mRNA |
| hsa-miR-424-5p | miRNA | CCND2 | mRNA |
| hsa-miR-424-5p | miRNA | PCDH17 | mRNA |
| hsa-miR-424-5p | miRNA | VEGFA | mRNA |
| hsa-miR-424-5p | miRNA | SLIT2 | mRNA |
| hsa-miR-424-5p | miRNA | DCLK1 | mRNA |
| hsa-miR-424-5p | miRNA | HSPG2 | mRNA |
| hsa-miR-424-5p | miRNA | TRANK1 | mRNA |
| hsa-miR-424-5p | miRNA | LURAP1L | mRNA |
| hsa-miR-424-5p | miRNA | MYB | mRNA |
| hsa-miR-424-5p | miRNA | RNF217 | mRNA |
| hsa-miR-424-5p | miRNA | PHF19 | mRNA |
| hsa-miR-424-5p | miRNA | CDCA4 | mRNA |
| hsa-miR-6838-5p | miRNA | SLC13A3 | mRNA |
| hsa-miR-6838-5p | miRNA | CEP55 | mRNA |
| hsa-miR-6838-5p | miRNA | TFAP2A | mRNA |
| hsa-miR-6838-5p | miRNA | CASR | mRNA |
| hsa-miR-6838-5p | miRNA | TNFSF13B | mRNA |
| hsa-miR-6838-5p | miRNA | MYBL1 | mRNA |
| hsa-miR-6838-5p | miRNA | KCNJ2 | mRNA |
| hsa-miR-6838-5p | miRNA | LAMP3 | mRNA |
| hsa-miR-6838-5p | miRNA | ARMH4 | mRNA |
| hsa-miR-6838-5p | miRNA | CCND2 | mRNA |
| hsa-miR-6838-5p | miRNA | PCDH17 | mRNA |
| hsa-miR-6838-5p | miRNA | VEGFA | mRNA |
| hsa-miR-6838-5p | miRNA | DCLK1 | mRNA |
| hsa-miR-6838-5p | miRNA | HSPG2 | mRNA |
| hsa-miR-6838-5p | miRNA | TRANK1 | mRNA |
| hsa-miR-6838-5p | miRNA | LURAP1L | mRNA |
| hsa-miR-6838-5p | miRNA | MYB | mRNA |
| hsa-miR-6838-5p | miRNA | RNF217 | mRNA |
| hsa-miR-6838-5p | miRNA | PHF19 | mRNA |
| hsa-miR-6838-5p | miRNA | CDCA4 | mRNA |
| hsa-miR-497-5p | miRNA | SLC13A3 | mRNA |
| hsa-miR-497-5p | miRNA | CEP55 | mRNA |
| hsa-miR-497-5p | miRNA | TFAP2A | mRNA |
| hsa-miR-497-5p | miRNA | CASR | mRNA |
| hsa-miR-497-5p | miRNA | TNFSF13B | mRNA |
| hsa-miR-497-5p | miRNA | MYBL1 | mRNA |
| hsa-miR-497-5p | miRNA | KCNJ2 | mRNA |
| hsa-miR-497-5p | miRNA | LAMP3 | mRNA |
| hsa-miR-497-5p | miRNA | ARMH4 | mRNA |
| hsa-miR-497-5p | miRNA | CCND2 | mRNA |
| hsa-miR-497-5p | miRNA | PCDH17 | mRNA |
| hsa-miR-497-5p | miRNA | VEGFA | mRNA |
| hsa-miR-497-5p | miRNA | DCLK1 | mRNA |
| hsa-miR-497-5p | miRNA | HSPG2 | mRNA |
| hsa-miR-497-5p | miRNA | TRANK1 | mRNA |
| hsa-miR-497-5p | miRNA | LURAP1L | mRNA |
| hsa-miR-497-5p | miRNA | MYB | mRNA |
| hsa-miR-497-5p | miRNA | RNF217 | mRNA |
| hsa-miR-497-5p | miRNA | PHF19 | mRNA |
| hsa-miR-497-5p | miRNA | CDCA4 | mRNA |
